# Effective exchange to a larger size catheter for a lung abscess with initial percutaneous drainage failure: a case report

**DOI:** 10.1186/s40792-020-00828-7

**Published:** 2020-04-03

**Authors:** Maki Oh, Shohei Mori, Yuki Noda, Daiki Kato, Takashi Ohtsuka

**Affiliations:** Department of Surgery, Division of Thoracic surgery, The Jikei University School of Medicine, 3-25-8 Nishishinbashi, Minatoku, Tokyo, 105-0003 Japan

**Keywords:** Lung abscess, Drainage, Catheter

## Abstract

**Background:**

Percutaneous catheter drainage is an effective therapy for antibiotic-refractory lung abscesses. Pulmonary resection is usually considered in cases of drainage failure, although it remains controversial.

**Case presentation:**

A 42-year-old man with antibiotic-refractory lung abscess underwent percutaneous abscess drainage with a 10-Fr pigtail catheter. However, adequate evacuation of the abscess content was not achieved, and his respiratory condition worsened and he required a ventilator. To achieve and maintain effective drainage, insertion of a larger size 28-Fr catheter to replace the 10-Fr catheter was performed under general anesthesia and one-lung ventilation with a double-lumen tube to isolate the left lung. Exchange with a larger size catheter was effective and achieved adequate drainage. The procedure was performed safely by expanding the route of the old catheter as a guide for accessing the abscess cavity. His condition immediately improved and he was discharged on day 40 post-catheter exchange with no complications and cured with a small residual thin wall cavity.

**Conclusions:**

Small size catheters are generally recommended for initial percutaneous drainage; however, we argue that exchange with larger size catheters should be primarily considered instead of pulmonary resection in cases of initial drainage failure. This may avoid the need for pulmonary resection.

## Background

Approximately 80−90% of lung abscesses are treated successfully with antibiotics; however, conservative medical therapy may occasionally fail due to various factors, thereby requiring surgical intervention such as percutaneous catheter drainage or pulmonary resection [1, 2]. The success rate of percutaneous catheter drainage is high [2–4]; however, it remains unclear whether pulmonary resection is an appropriate alternative in cases of initial drainage failure.

## Case presentation

**Fig. 1:**
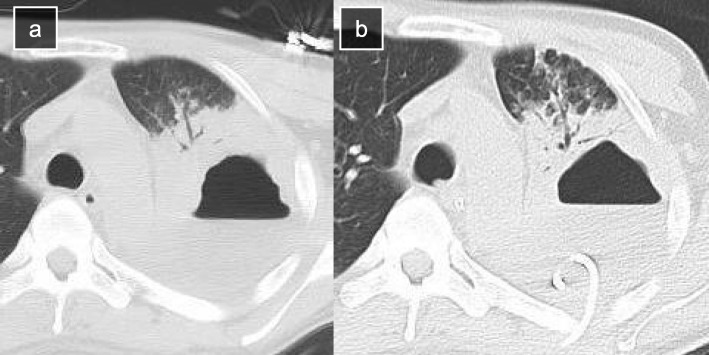
Computed tomography findings before and after initial percutaneous drainage. **a** A large abscess with an air-fluid level in the left upper lobe. **b** Residual abscess content due to incomplete initial drainage

**Fig. 2:**
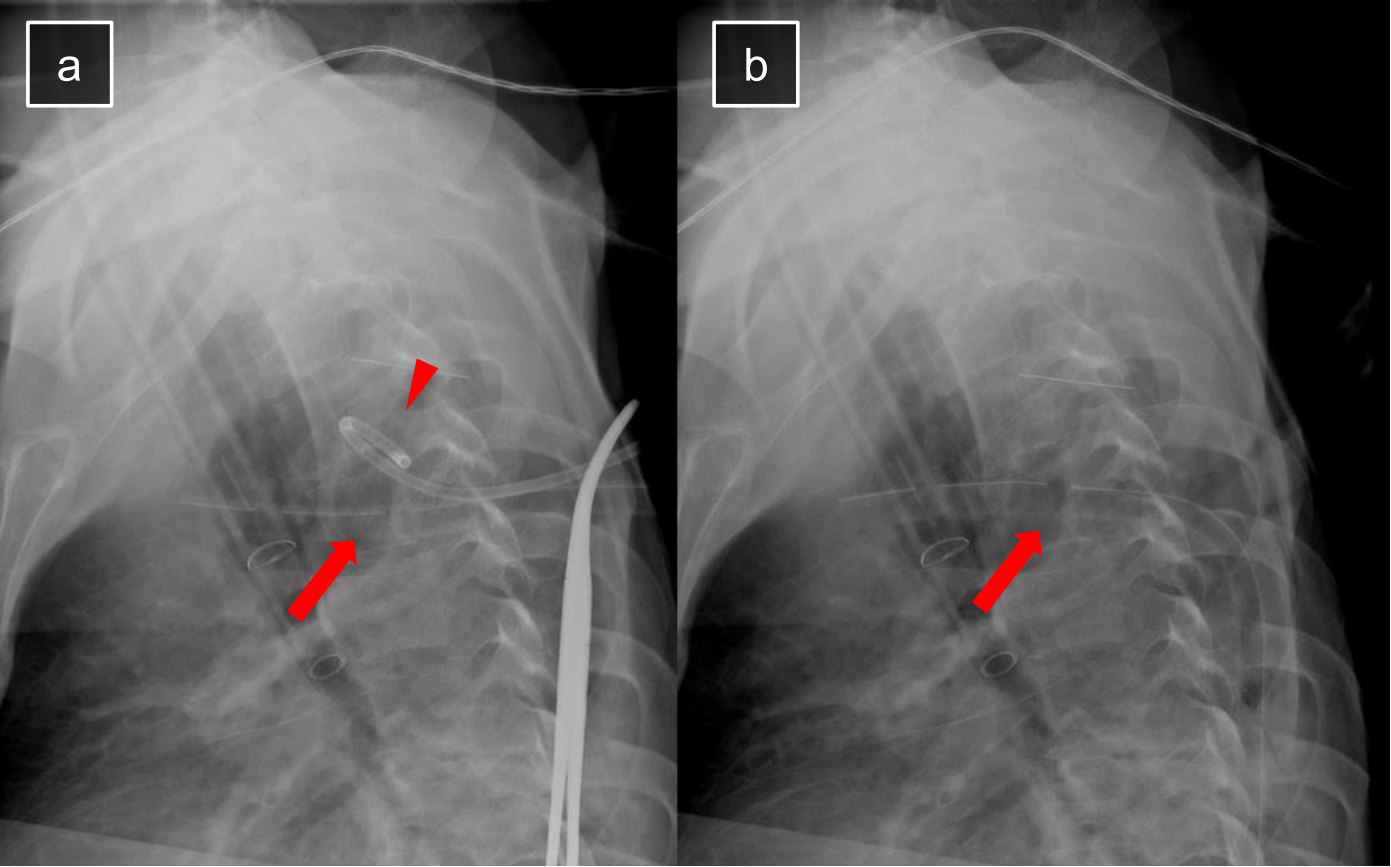
Catheter exchange procedure. **a** The 10-Fr pigtail catheter (arrowhead) is used as a guide for accessing the abscess cavity. **b** Exchange with a larger size (28 Fr) catheter (arrow)

A 42-year-old man is presented with complaints of persistent fever and left chest pain since 2 weeks. He had no remarkable medical history. Chest radiograph showed diffuse consolidation of the left lung. Chest computed tomography (CT) revealed a large abscess with an air-fluid level in the left upper lobe and extensive consolidation of the whole left lung (Fig. 1a). He was diagnosed with severe pneumonia with a lung abscess. Antibiotic therapy was started empirically with meropenem and vancomycin. Antibiotic therapy administered for 5 days did not demonstrate a satisfactory clinical outcome. Thereafter, he underwent CT-guided percutaneous catheter drainage of the lung abscess with a 1-Fr pigtail catheter, and 42 mL of pus fluid was evacuated. However, the catheter became clogged the next day, after which the patient developed respiratory failure and required a ventilator. Chest CT revealed pneumonia of the right lung and residual abscess content due to incomplete initial drainage (Fig. 1b). We suspected that pneumonia of the right lung was caused by aspiration of the abscess content due to the right lateral position of the patient at the time of the CT-guided percutaneous catheter drainage. Frequent bronchoscopies were performed to remove the endobronchial pus; however, his respiratory failure worsened further and he underwent tracheostomy. To achieve and maintain effective drainage, insertion of a larger size catheter to replace the 10-Fr catheter was planned. Since the route was expanded sufficiently without a lung trauma, large enough catheter, 28 Fr, was chosen as a new one in favor of avoiding clogging. The procedure of exchange of catheter was performed under general anesthesia and one-lung ventilation with a double-lumen tube to isolate the left lung and prevent aspiration of abscess content to the right lung due to the right lateral position of the patient. Expansion of the route reaching the abscess cavity was safely performed by utilizing the old catheter as a guide; additional skin incision of 2 cm was made at the insertion point of the old catheter, and subcutaneous tissue and muscle layers around the catheter were cut with an electric scalpel, and the route was expanded with a straight Pean forceps inserted along the catheter, confirming the tip of the forceps to be reaching to the cavity by x-ray fluoroscopy. Since the route was expanded sufficiently without a lung trauma, enough large catheter, 28 Fr, was chosen as a new one in favor of avoiding occlusion. After placing the new catheter into the cavity, the old one was removed (Fig. 2a, b). The new catheter was led out to a new wound through a subcutaneous tunnel.

**Fig. 3:**
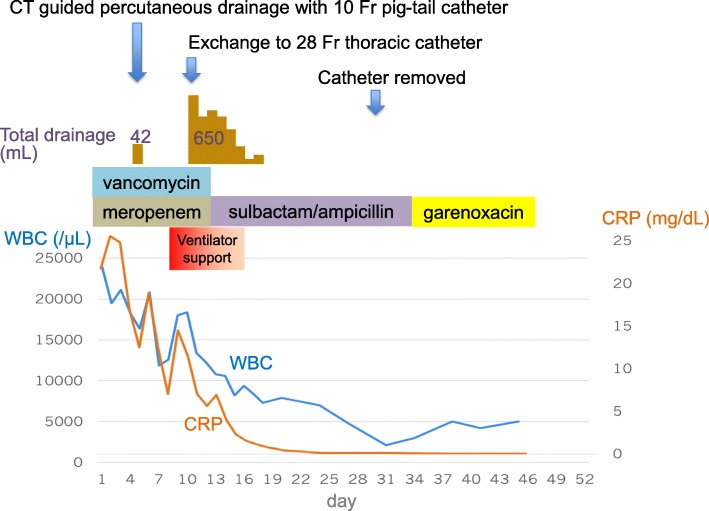
Clinical course of this case. The patient’s respiratory condition and laboratory examination results immediately improved; he was free from ventilator support on day 5 post-catheter exchange. The catheter was removed on day 18 post-catheter exchange

**Fig. 4:**
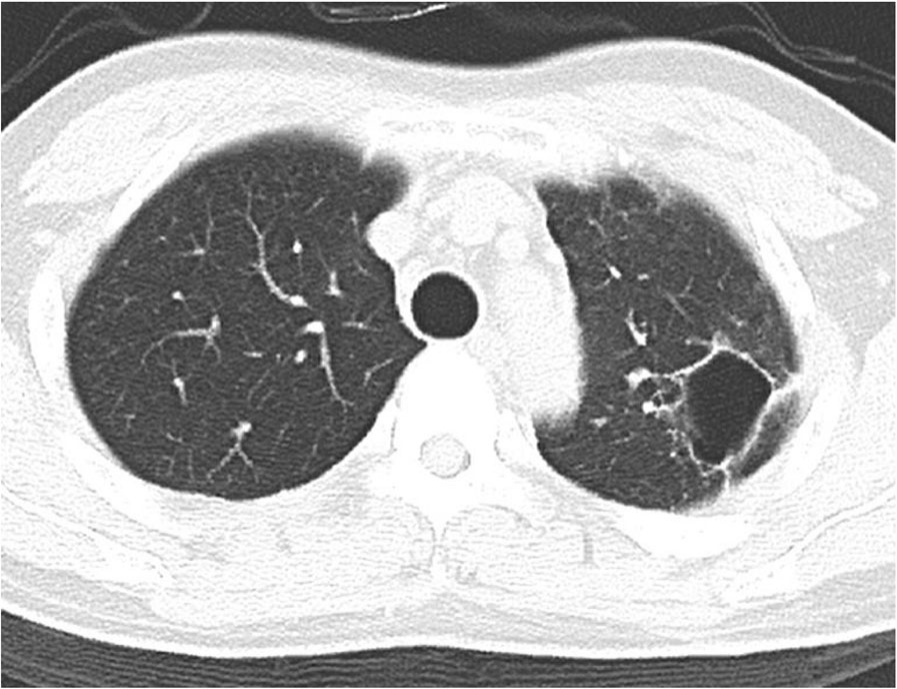
Computed tomography findings after 7 months. The lung abscess is completely cured with a small residual thin wall cavity

The larger sized catheter enabled the maintenance of adequate drainage of a total of 650 mL for 8 days. A suction pressure was set at − 10 cmH_2_O. Although air leakage from the drainage catheter continued until the catheter was removed, it did not cause any clinical problems. The antibiotic therapy was de-escalated to sulbactam sodium/ampicillin sodium owing to identification of the causative bacteria from a culture of the initial evacuated pus fluid; *Prevotella buccae*, *Streptococcus anginosus*, *and Fusobacterium necrophorum* were detected. His respiratory condition and laboratory results immediately improved, and he was free of ventilator support on day 5 post-catheter exchange. The catheter was removed on day 18. In addition, the antibiotic therapy was switched to oral garenoxacin and discontinued on day 35. He was discharged on day 40 post-catheter exchange with no complications. Figure 3 shows the clinical course of this case. After 7 months, his chest CT confirmed complete cure of the lung abscess, albeit with only a small residual thin wall cavity (Fig. 4).

## Discussion

The clinical course of this case provides two important suggestions. Exchange of a small size catheter with that of a larger size can be effective for antibiotic-refractory lung abscesses when the initial abscess drainage fails. The exchange procedure can be performed safely by isolating the affected lung to prevent the aspiration of abscess content and utilizing the old catheter as a guide for accessing the abscess cavity.

First, exchange to a larger size catheter can be effective for lung abscesses when the initial abscess drainage by small size catheter fails. Although percutaneous catheter drainage is an effective treatment alternative with a higher success rate than pulmonary resection for antibiotic-refractory lung abscesses, it occasionally fails due to catheter clogging with tenacious and viscous material [2, 5, 6]. The ideal catheter size for abscess drainage is not well established. It has been reported that a small size (10–14 Fr) catheter enables adequate and effective drainage [2, 7]. Furthermore, the use of a larger size catheter is considered unnecessary because it may cause undesired trauma to the lung and lead to pneumothorax, hemothorax, empyema, and bronchopleural fistula [6]. On the other hand, pulmonary resection, considered in cases of percutaneous drainage failure, yields complications and high mortality rates [2, 8]. So, we argue that a small size catheter should be chosen for initial percutaneous drainage; however, exchange with a larger size catheter should be considered as the primary treatment instead of pulmonary resection, in cases of initial drainage failure. Regarding the size of exchange, the larger drain, the higher risk of lung trauma, but in this case, if the drain excahnge had failed, a lung resection would be required. Because the route was safely expanded enough, a sufficient large size, 28 Fr, was selected to avoid clogging. However, a smaller size drain, such as 18 or 20 Fr, may have been enough effective.

The catheter exchange was performed after the respiratory condition worsened, but it might not be an optimal timing. When only a small amount was evacuated and the catheter was clogged the next day, immediate catheter exchange might be able to avoid ventilator support.

Second, the exchange procedure can be performed safely by isolation of the affected lung to prevent aspiration and utilizing the old catheter as a guide for reaching the abscess cavity. Aspiration of abscess content to the contralateral lung because of a change in the patient’s position can be a trigger for worsening of the respiratory condition [1]. In our case, we should have performed an initial CT-guided percutaneous drainage under lung isolation or restriction of the positional change. Also, CT-guided drainage in a supine position should have been considered to avoid aspiration pneumonia to the contralateral lung. Therefore, we performed the catheter exchange procedure under general anesthesia and one-lung ventilation with a double-lumen tube to isolate the affected lung and prevent aspiration. We believe that this method was successful because the patient’s respiratory condition did not worsen further. In addition, using the old catheter as a guide for access into the abscess cavity allowed safe expansion of the route necessary for insertion of a large size catheter without any undesirable lung injury.

## Conclusions

Exchange of a small size catheter with that of a larger size was effective for lung abscesses with initial abscess drainage failure. This procedure could be performed safely by using the old catheter as a guide for accessing the abscess cavity. We suggest that exchange with a larger size catheter should be primarily considered instead of pulmonary resection in cases of initial drainage failure.

## Data Availability

Not applicable.

## Abbreviation

CT: Computed tomography
